# *M.tuberculosis* Mutants Lacking Oxygenated Mycolates Show Increased Immunogenicity and Protective Efficacy as Compared to *M. bovis* BCG Vaccine in an Experimental Mouse Model

**DOI:** 10.1371/journal.pone.0076442

**Published:** 2013-10-17

**Authors:** Dorsaf Hedhli, Olivier Denis, Daniel Barkan, Mamadou Daffé, Michael S. Glickman, Kris Huygen

**Affiliations:** 1 Service Immunology, Scientific Institute of Public Health (WIV-ISP, Site Ukkel), Brussels, Belgium; 2 Immunology Program and Division of Infectious Diseases, Memorial Sloan Kettering Cancer Center, New York, New York, United States of America; 3 Département de Mécanismes Moléculaires des Infections Mycobactériennes, Institut de Pharmacologie et Biologie Structurale du CNRS et de l'Université Paul Sabatier (UMR 5089), Toulouse, France; University of Palermo, Italy

## Abstract

The existing vaccine against tuberculosis (*M. bovis* BCG) exerts some protection against the extrapulmonary forms of the disease, particularly in young children, but is not very effective against the pulmonary form of TB, which often results from the reactivation of a latent *M. tuberculosis* (*M.tb*)infection. Among the new approaches in TB vaccine development, live attenuated *M.tb* mutants are a promising new avenue. Here we report on the vaccine potential of two highly attenuated *M.tb* mutants, MGM1991 and *M*.*tb*hma::hyg (HMA), lacking all oxygenated mycolates in their cell wall. In C57BL/6 mice, stronger Th1 (IFN-γ, IL-2 and TNF-α) and IL-17 responses could be induced following subcutaneous vaccination with either of the two mutants, than following vaccination with *M. bovis* BCG. Significantly more mycobacteria specific IFN-γ producing CD4^+^ and particularly CD8^+^ T cells could be detected by intracellular cytokine staining in mice vaccinated with the *M.tb* mutants. Finally, vaccination with either of the two mutants conferred stronger protection against intratracheal *M.tb* challenge than vaccination with BCG, as indicated by reduced bacterial replication in lungs at 4 to 12 weeks after challenge. Protection against *M. tb* dissemination, as indicated by reduced bacterial numbers in spleen, was comparable for both mutants to protection conferred by BCG.

## Introduction

A better understanding of the protective immune response against *M.tb* is very important in the quest for better TB vaccines, but the actual “correlates of protection” are unknown so far and will perhaps only be identified from successful vaccine trials [1]. A major role in protection against this intracellular pathogen is played by the cellular arm of the adaptive immune system, particularly by CD4^+^ Th1 type T helper cells producing IFN-γ, TNF-α and IL-2. This is underscored by the clinical association between HIV and TB [2] and by the increased risk to develop reactivation TB of individuals treated with anti-TNF-α agents used for a range of inflammatory/autoimmune diseases, such as rheumatoid arthritis and Crohn’s disease [3]. Besides MHC class II restricted CD4^+^ T cells also MHC class I restricted CD8^+^ T cells play an important role in the immune response against *M.tb* through production of cytokines and their lytic activity targeting infected cells. This has been demonstrated by a number of studies in preclinical models [4] and with samples isolated from humans [5]. The importance of CD8^+^ T cells in the control of latent TB infection and immune protection against reactivation TB in humans was convincingly demonstrated by Bruns et al, who have reported that anti-TNF-α immunotherapy with infliximab (a monoclonal antibody against tumour necrosis factor a (TNF-a) used to treat autoimmune diseases) reduced CD8^+^ T cell-mediated antimicrobial activity against *M.tb* in humans through the interaction of the antibody with cell surface TNF on the CD8^+^ T cell and their subsequent complement-mediated lysis [6]. Clearly both CD4^+^ and CD8^+^ T cells play an important role in protective immunity against *M*.*tb*.

It is well known that BCG vaccination is a weak inducer of CD8^+^ T cells as compared to tuberculosis infection and a 200-fold higher dose of BCG is needed to induce CD8^+^ responses comparable to those induced with *M.tb* [7]. This weak CD8^+^ generating potential may be a major reason for the failure of BCG as a vaccine [1] and besides displaying a broader antigenic repertoire, live, attenuated *M.tb* mutants could offer the advantage over BCG of inducing strong CD8^+^ responses. Here we report on the analysis of the vaccine potential of two *M.tb* mutants, targeted in their cell wall composition, specifically their mycolic acid composition. *M.tb* has three classes of mycolic acid, the α-mycolates and two oxygenated forms the methoxy- and ketomycolates respectively. These major lipids of the *M.tb* cell wall, are modified by cyclopropane rings, methyl branches, and oxygenation through the action of eight mycolic acid methyl transferases (MAMTs), located in 4 genetic loci. By sequentially deleting all functional MAMTs from the *M.tb* strain Erdman chromosome and analyzing these strains *in vitro* and *in vivo*, we have previously shown that the MGM1991*M*.*tb* mutant with only α-mycolates without cyclopropane rings, is viable but severely attenuated after aerosol infection in mice [8]. Another *M.tb* mutant , i.e. *M*.*tb*hma::hyg (HMA) with inactivated *hma* (also called *MmaA4*) gene (as MGM1991 lacking all oxygenated mycolic acids, but with α-mycolates with distal and proximal cyclopropane rings) was also found to be significantly attenuated *in vivo* for growth in a mouse model, despite comparable *in vitro* growth rate in THP-1 cells as parental*M*.*tb* H37Rv strain[9]. 

Here, we report on the analysis of the vaccine potential of these two mutants lacking all oxygenated mycolic acids. C57BL/6 mice were vaccinated subcutaneously with *M.tb* MGM1991 or *M.tb* HMA mutant or with *M. bovis* BCG vaccine and immunogenicity and protective efficacy against virulent *M.tb* was compared for the three strains.

## Materials & Methods

### Ethics statement

All experiments were reviewed and approved by the ethical committee of the Institute of Public Health–Veterinary and Agrochemical Research Institute (WIV-ISP/CODA-CERVA; Brussels, Belgium). 

### Mice

C57BL/6, BALB/c and DBA/2 mice were bred in the animal facilities of the Scientific Institute of Public Health (Brussels, Belgium) from breeding pairs obtained from Harlan laboratories (The Netherlands). Mice were 6–8 weeks of age at the start of experiments. 

### Construction of *M. tuberculosis* mutants

Details concerning mutant constructions were previously described by Barkan*etal*[8] and Dubnau*etal*[9]. *M.tb* mutant MGM1991 (Erdman strain, Δ*mmaA1-4*::*loxPattB*::(*strep*), Δ *pcaA/umaA1*::*hyg*; D*cmaA2*::*zeo*) lacks all oxygenated (keto and methoxy) mycolates and its α-mycolate lacks the distal and proximal cyclopropanation[8]. For the construction of *M. tuberculosis hma::hyg* (HMA) mutant, chromosomal *hma* (*mmaA4*)gene of *M.tb* H37Rv was inactivated by allelic replacement resulting in a strain lacking all oxygenated (keto and methoxy) mycolates, but with distal and proximal cyclopropanation of its α-mycolate [9]. 

### Demonstration of attenuated phenotype of MGM1991 mutant

C57BL/6 mice were infected by the intratracheal route with 10^4^ CFU of either parental *M.tb* Erdman strain or MGM1991 mutant and bacterial replication in lungs was monitored for 70 days. DBA/2 mice were infected by the intratracheal route with 10^5^ CFU of either parental *M.tb* Erdman strain or MGM1991 mutant and monitored in a long term survival experiment for 40 weeks. 

### MGM1991, HMA and BCGvaccination experiments

M.*tb* MGM1991 and *M.tb* hma::hyg (HMA) were grown on liquid 7H9 medium supplemented with oleic acid-albumin-dextrose-catalase to an O.D. of 0.4-0.6. Bacterial stocks were kept frozen in 20% glycerol at -70°C until use. Bacterial CFU numbers were determined by plating serial dilutions on 7H11 Middlebrook agar supplemented with oleic acid-albumin-dextrose-catalase.

*M. bovis* BCG (strain GL2, derived from 1173P2 Pasteur strain) and luminescent *M.tb* H37Rv were grown as a surface pellicle on synthetic Sauton medium as described before[10]. Bacteria were harvested after 2 weeks, bacterial pellicle homogenized using ball mill and aliquots were stored frozen at 70°C in 20% glycerol until use.

A dose of 5x10^4^ CFU (C57BL/6) or 5x10^5^ CFU (BALB/c) of MGM1991, HMA or BCG was inoculated subcutaneously in the left flank.

### Cytokine production

At day 30 and day 140 post-immunization, vaccinated mice were sacrificed and spleen and inguinal lymph nodes (day 30 only) were removed aseptically. Organs were homogenized using a loosely fitting Dounce homogenizer and leukocytes were cultured at 4x10^6^ cells/ml in RPMI-1640 medium supplemented with 10% FCS, penicillin and streptomycin and 5x10^-5^ M 2-mercapto-ethanol, in round-bottom microwell plates. Spleen cells from five mice per group were tested for cytokine response to PPD (Purified protein derivative 25 µg/ml) prepared from *M.tb*, recombinant *E. coli* derived Ag85A (Rv3804c, 10 µg/ml), Ag85A _241-260_10 µg/ml (immunodominant I-A^b^ restricted peptide of Ag85A (Rv3804c) described by D'Souza S *etal* 2003[11]), ESAT6-_1-20_ peptide 10 µg/ml (immunodominant I-A^b^ peptide of early secretary antigenic target-6 peptide [12]or recombinant *E. coli* derived DosR antigens Rv1733c, Rv2626c, Rv2627c and Rv2628 (10 µg/ml)[13]. Supernatants were harvested after 24 hr (IL-2 and TNF-α) or 72 h (IFN-γ and IL-17A) when peak values of the respective cytokines can be measured. Spleen cells were tested individually for cytokine response whereas lymph node cells were pooled. Supernatant were stored frozen at −20 °C until analysis. IFN-γ was detected using ELISA with purified rat anti-mouse IFN-γ as capture and biotin rat anti-mouse IFN-γ as detection antibody (BD Pharmingen), IL-17Aand TNF-α were detected by commercial ELISAs kit (e-Bioscience) (IL-17A detection level: 4 pg/ml, IFN-γ detection level: 5 pg/ml, TNF-α detection level: 8 pg/ml). 

### IFN-γ and IL-17A enzyme-linked immunospot (ELISPOT) assay

Specific spleen cell IFN-γ secretion was assayed by ELISPOT as described by Romano et al[14]. Briefly, 96-well surfactant-free mixed cellulose ester membrane plate (Millipore) were coated overnight at 4°C with 50 l of capture purified anti-mouse IFN-γ in phosphate-buffered saline (PBS 15 g/ml; BD Pharmingen) and then saturated with RPMI-1640 medium (Gibco, Grand Island, NY) supplemented with penicillin, streptomycin, 5×10^−5^M 2-mercaptoethanol, 10% FCS for 2 hr at 37°C. Lung leukocytes (pool of five mice per group) were added at a known concentration in the same medium in the presence or absence of peptides (5 or 10 µg/ml) and plates were incubated for 40 h at 37°C and 5% CO_2_. After extensive washing, plates were incubated overnight at 4°C with 50 µl of biotinylated rat anti-mouse IFN-γ (2 g/ml) (BD Pharmingen), washed and incubated for 45 min at 37°C and 5% CO_2_ with alkaline phosphatase-labelled streptavidin (Sigma). After washing, spots were revealed with Bio-Rad (Hercules, CA) alkaline phosphatase conjugate substrate kit, following the manufacturer's instructions and plates were analysed on a Bioreader 3000 LC (BioSys, Germany). IL-17 enzyme-linked immunospot (ELISPOT) assay was performed in the same manner with Purified rat anti-mouse IL-17A as a capture antibody (BD Pharmingen) and Biotin rat anti-mouse IL-17A as a detection antibody (BD Pharmingen).

Results are shown as mean spot-forming cells (SFC) per million leukocytes.

### Intracellular cytokine staining

For intracellular cytokine staining, cell suspensions of splenocytes were stimulated with 25 µg/ml of *M.tb* culture filtrate for 5 days, purified on Ficoll and restimulated a second time during 24h and treated with 10 µg/ml of Brefeldin A for 4 h at 37°C with 5% CO2. After this incubation period, cells were marked, fixed and permeabilized for intracellular staining. The presence of intracellular IFN-γ was determined by using anti-IFN-γ (cloneXMG1.2, eBiosciences). Cells were further labelled with a fluorochrome-labeled monoclonal antibody specific for CD4 (clone RM4-5) or CD8a (53-6.7). Cells were analysed using CELLQuest software on a Becton Dickinson FACSCalibur flow cytometer. 

### *M. tuberculosis* challenge

12 weeks after vaccination, mice were infected by intratracheal instillation with 5x 10^3^ or 5x10^4^ RLU (relative light units) of luminescent *M.tb* H37Rv corresponding to 10^4^ and 10^5^ CFU respectively [15]. Mycobacterial load in lungs and spleen of *M.tb* infected mice was quantified using a bioluminescence assay (determination of relative light units RLU) at 4 and 12 weeks after infection [10]. In this assay, RLU data correlate with CFU data as the luminometric assay only detects living bacteria [16].

## Results 

### *M. tuberculosis* mutants MGM1991 and HMA do not replicate *in vivo* following subcutaneous injection

**Figure 1:**
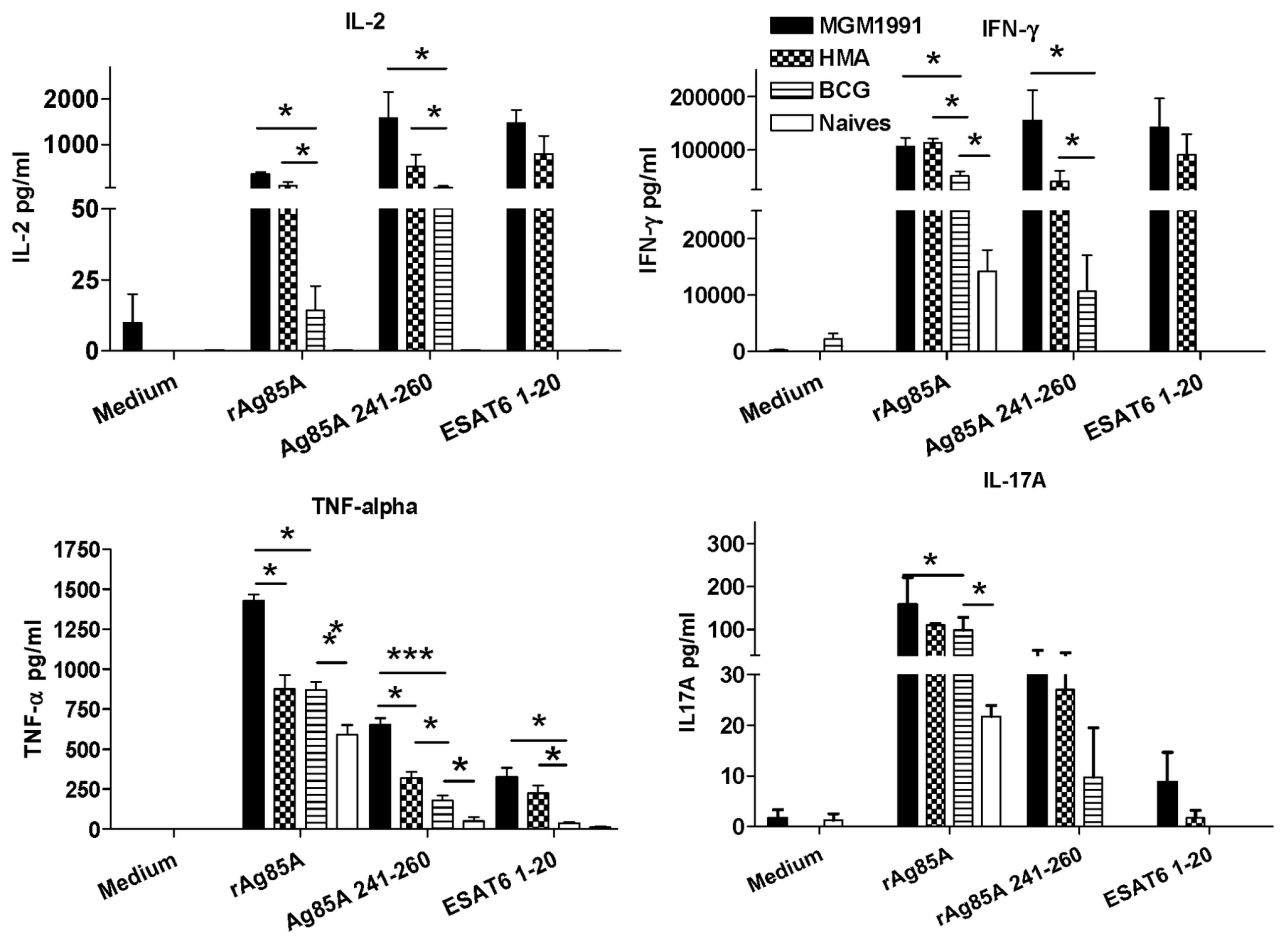
Spleen cell cytokine production in mice vaccinated with MGM1991, HMA or BCG vaccine. IL-2, IFN-γ, TNF-α and IL-17A levels in spleen cell culture supernatant of C57BL/6 mice vaccinated 4 weeks before with 5x10^4^ CFU of MGM1991, HMA or *M. bovis* BCG by the subcutaneous route or from unvaccinated (naïve) mice and stimulated in vitro with recombinant Ag85A (5 µg/ml) or with I-A^b^ restricted immunodominant peptides spanning aa 241-260 of Ag85A or aa 1-20 of ESAT-6 (10 µg/ml). Cytokine levels are expressed in pg/ml (mean ± SD of 5 mice tested individually). * *p* < 0.05; *** *p* < 0.001; (Mann-Whitney test).

We previously demonstrated the importance of oxygenated mycolic acids for virulence of *M.tb* in mice, using low-dose aerosol infection. Mice infected with *M.tb* HMA mutant showed a one hundred-fold reduced bacterial load in lungs up to 32 weeks after low-dose aerosol infection as compared to mice infected with parental *M.tb* strain H37Rv [9]. Likewise, mice infected by low-dose aerosol with mutant MGM1995 (which is identical to mutant MGM1991 used in this study) showed a fifty-fold lower CFU count in lungs over a period of 130 days than mice infected with parental MGM 1985 *M.tb* strain[8]. Finally, a dose of 10^4^ CFU of MGM1991 mutant administered by the intratracheal route resulted in a more than four hundred fold reduction in lung CFU counts on day 70 as compared to a same dose of parental *M.tb* strain Erdman (3,78 ± 0,7 log_10_ CFU versus 6,46 ± 0,27 log_10_ CFU; Dlog10 = 2.68) (**Left panel** Figure **1**). In another experiment, a dose of 10^5^ CFU/mice was used to infect highly sensitive DBA/2 mice by the intratracheal route for a survival study. Mice infected with WT *M.tb* strain (Erdman) showed a median survival time of 7 weeks whereas mice infected with MGM1991 *M.tb* mutant showed a median survival time of 29 weeks (**Right panel** Figure **1**). Before testing the vaccine potential of the *M. tb* mutants, we first examined the safety profile of a subcutaneous administration of 5x10^4^ CFU of the two *M.tb* mutants, the *M. bovis* BCG vaccine or parental *M. tuberculosis* H37Rv over a period of 20 weeks. Bacterial numbers in spleen and lungs were very low or below detection level (1.5 log_10_ CFU), whereas some bacteria could be recovered till week 6 from draining lymph nodes of mice vaccinated with either of the two *M. tb* mutants or *M. bovis* BCG (Table **1**). *M.tb* H37Rv levels were also very low 20 weeks after subcutaneous infection. Therefore, the subcutaneous route was choosen for all further vaccination experiments.

**Table 1:** Bacterial load in lungs, spleen and inguinal lymph nodes at weeks 1, 6 and 20 after subcutanous immunization with MGM1991, HMA, *M. bovis* BCG or *M.tuberculosis* H37Rv.

Mean Log_10_ CFU/organ									
Group	Week 1			Week 6			Week 20		
	Lung	Spleen	Lymph node	Lung	Spleen	Lymph node	Lung	Spleen	Lymph node
MGM1991	Nd	1,6±0,14	2,26 (2)	2±0,49	2,5±0,59	3,53 (3)	Nd	Nd	Nd (3)
HMA	Nd	Nd	1,82 (2)	-	1,8±0,28	3,27 (3)	1,8±0,6	2±0,6	Nd (4)
BCG	Nd	Nd	3,31 (2)	Nd	Nd	1,52 (3)	Nd	Nd	Nd (4)
H37Rv							2,1±0,58	Nd	2,3 (3)

Safety profile of *M.tb* mutant strains was examined after subcutaneous administration of 5x10^4^ CFU of the two *M.tb* mutants, the *M. bovis* BCG vaccine or parental *M. tuberculosis* H37Rv. Bacterial load was monitored in lungs, spleen and inguinal lymph nodes over a period of 20 weeks. Results are reported as mean log_10_ colony forming unit (CFU) ± SD/total lungs or spleen (2 to 4 mice tested individually) or/lymph nodes (pooled organs from 2 to 4 animals/group), Nd: not detected.

### Vaccination with *M. tuberculosis* MGM1991 and HMA mutants stimulates stronger mycobacteria specific Th1 and Th17 type cytokine responses than vaccination with *M. bovis*BCG.

C57BL/6 mice were sacrificed 30 days after subcutaneous vaccination with 5x10^4^ CFU of *M.tb* mutants or *M. bovis* BCG. Spleen cells were stimulated *in vitro* with mycobacterial antigens and culture supernatants were analyzed for presence of Th1 cytokines IL-2, IFN-γ and TNF-α and Th17 cytokine IL-17A.

As shown in Figure **1**, significantly higher levels of the three cytokines were produced by spleen cells from mice vaccinated with *M.tb* mutants than following vaccination with BCG in response to the mycolyl transferase Ag85A (Rv3804c) and its immunodominant I-A^b^ restricted epitope present in the 20mer peptide spanning aa 241-260. As expected, the I-A^b^ restricted epitope of ESAT-6 (spanning aa 1-20) was only recognized by T cells from mice vaccinated with the *M.tb* mutants and not by T cells of mice vaccinated with BCG (which has a deletion in the RD1 region encoding ESAT-6). Whereas spleen cell IL-2 and IFN-γ levels induced with MGM1991 and HMA were of comparable magnitude, TNF-α responses were clearly higher in animals vaccinated with MGM1991 than with HMA mutant. Spleen cell IL-17A levels were only modestly increased, but tended to be higher in animals vaccinated with the two *M.tb* mutants than in animals vaccinated with BCG.

**Figure 2:**
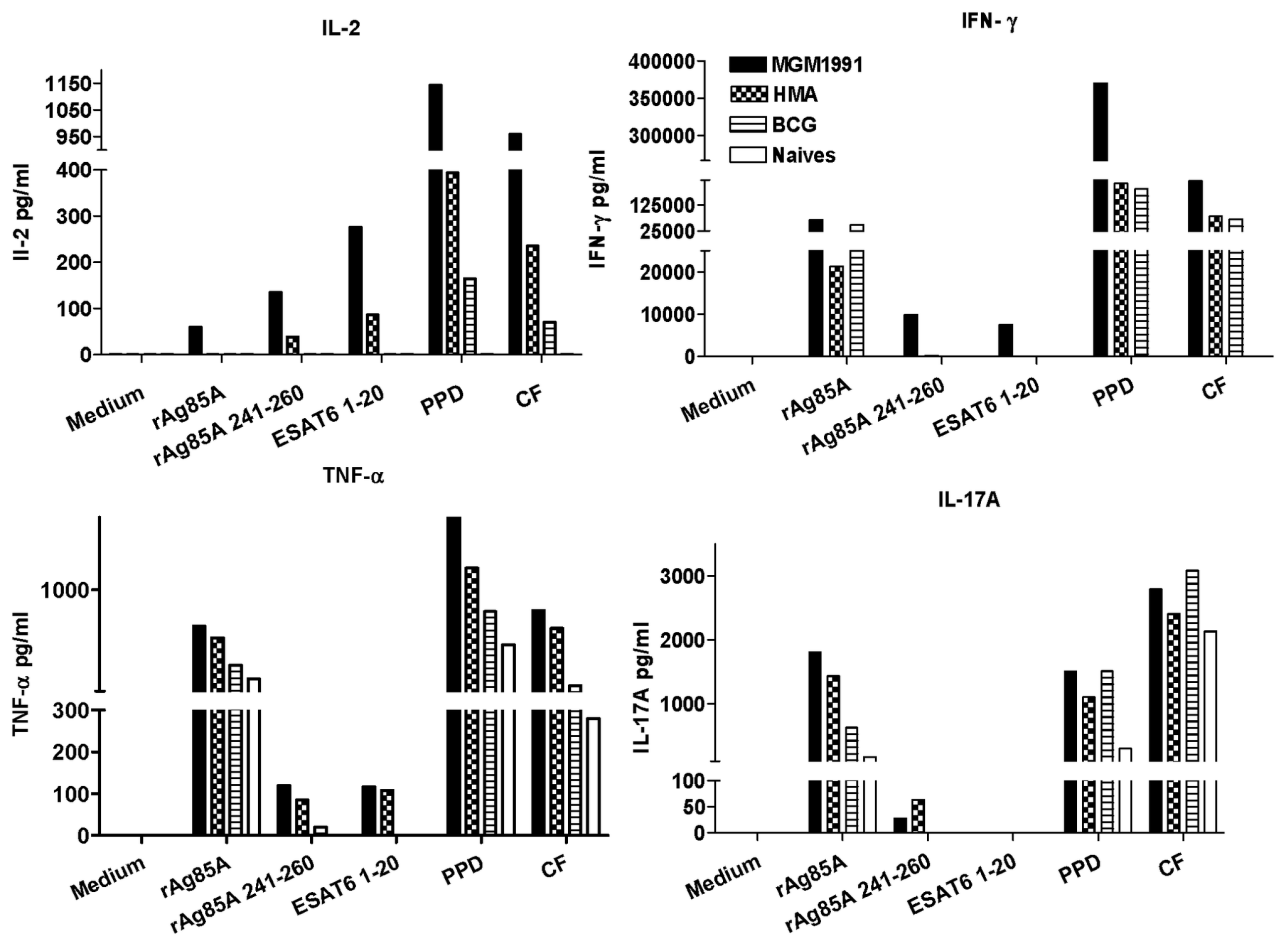
Cytokine production of draining lymph node cells of mice vaccinated with MGM1991, HMA or BCG vaccine. IL-2, IFN-γ TNF-α and IL-17A levels in culture supernatant of pooled inguinal lymph node cells of C57BL/6 mice vaccinated 4 weeks before with 5x10^4^ CFU of MGM1991, HMA or *M. bovis* BCG by the subcutaneous route or from unvaccinated (naïve) mice and stimulated in vitro with recombinant Ag85A (5 µg/ml), with I-A^b^ restricted immunodominant peptides spanning aa 241-260 of Ag85A or aa 1-20 of ESAT-6, with purified protein derivative PPD or with culture filtrate from *M.tb* culture filtrate CF (10 µg/ml). Cytokine levels are expressed in pg/ml (pooled cells from 5 animals/group).

Also in culture supernatant from pooled, inguinal lymph node cells (Figure **2**), higher IL-2, IFN-γ and TNF-α responses to mycobacterial antigens were detected in animals vaccinated with *M.tb* mutants MGM1991 and HMA than in animals vaccinated with BCG. Mycobacteria specific IL-2 and IFN-γ production was higher following vaccination with MGM1991 than with HMA mutant, whereas TNF-α levels were comparable in animals vaccinated with either *M.tb* mutant. IL-17A levels were clearly higher in draining lymph nodes than in spleen, and lymph node cells from mice vaccinated with the *M.tb* mutants produced more IL-17A upon stimulation with recombinant Ag85A and its immunodominant peptide Ag85A _241-260_ than lymph nodes cells from mice vaccinated with the BCG vaccine.

**Figure 3:**
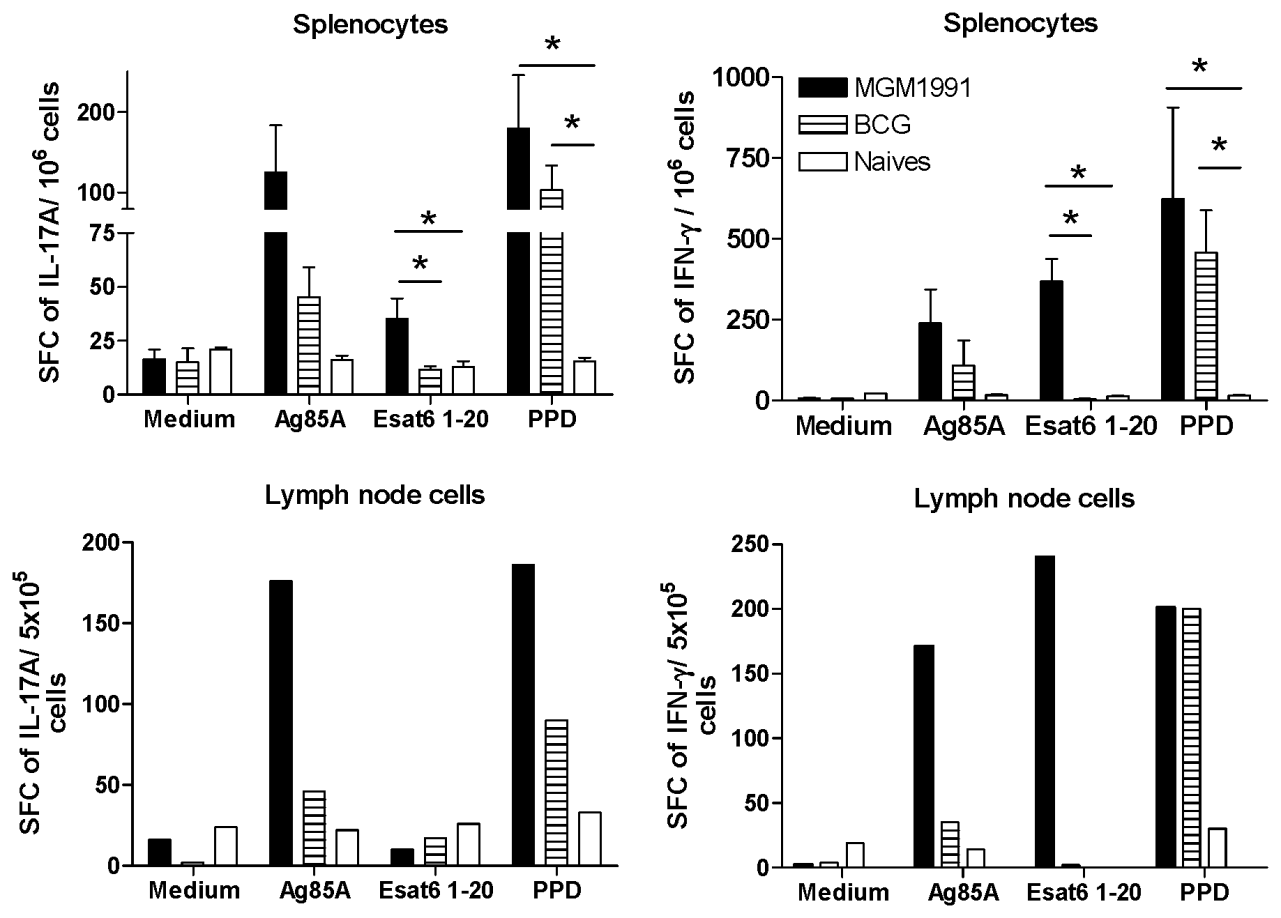
IFN-γ and IL-17A ELISPOT cells in spleen and draining lymph nodes of mice vaccinated with MGM1991 or BCG. IFN-γ and IL-17A producing cells in spleen and inguinal lymph nodes of C57BL/6 mice vaccinated 4 weeks before with 5x10^4^ CFU of MGM1991 or *M. bovis* BCG by the subcutaneous route or from unvaccinated (naïve) mice and stimulated in vitro with recombinant Ag85A (5 µg/ml), with I-A^b^ restricted immunodominant peptide spanning aa 1-20 of ESAT-6 or with purified protein derivative PPD from *M.tb* (10 µg/ml). Cytokine levels are expressed as mean number of spot forming cells/ 10^6^ spleen cells (5 mice tested individually) or as mean number of spot forming cells/ 5x10^5^ lymph node cells (pooled cells from 5 animals/group). * *p* < 0.05 (Mann-Whitney test).

In IL-17A and IFN-γ ELISPOT assays, spleen and pooled lymph node cells from MGM1991 vaccinated mice showed stronger Ag85A specific T cell responses than spleen or pooled lymph node cells from BCG vaccinated mice. ESAT-6 specific ELISPOT responses were only detected after vaccination with *M.tb* mutant MGM1991, not with BCG (Figure **3**)**.**

Mycobacteria-specific spleen cell IFN-γ responses were also analysed in C57BL/6 mice vaccinated 20 weeks before with 5x10^4^ CFU of MGM1991, HMA or BCG. Vaccination with MGM1991 mutant induced the strongest responses upon stimulation with PPD, Ag85A and ESAT-6. Low IFN-γ responses were detected following stimulation with either of four antigens encoded by the dormancy regulon DosR [13]. Responses to Rv1733c and Rv2628 were significantly higher in spleen from MGM1991 vaccinated mice than in spleen from mice vaccinated with HMA or BCG or from naïve mice (Table **1**)

### Vaccination with *M. tuberculosis* MGM1991 and HMA mutants stimulates stronger mycobacteria specific CD4^+^ and CD8^+^ response than vaccination with *M. bovis* BCG

**Figure 4:**
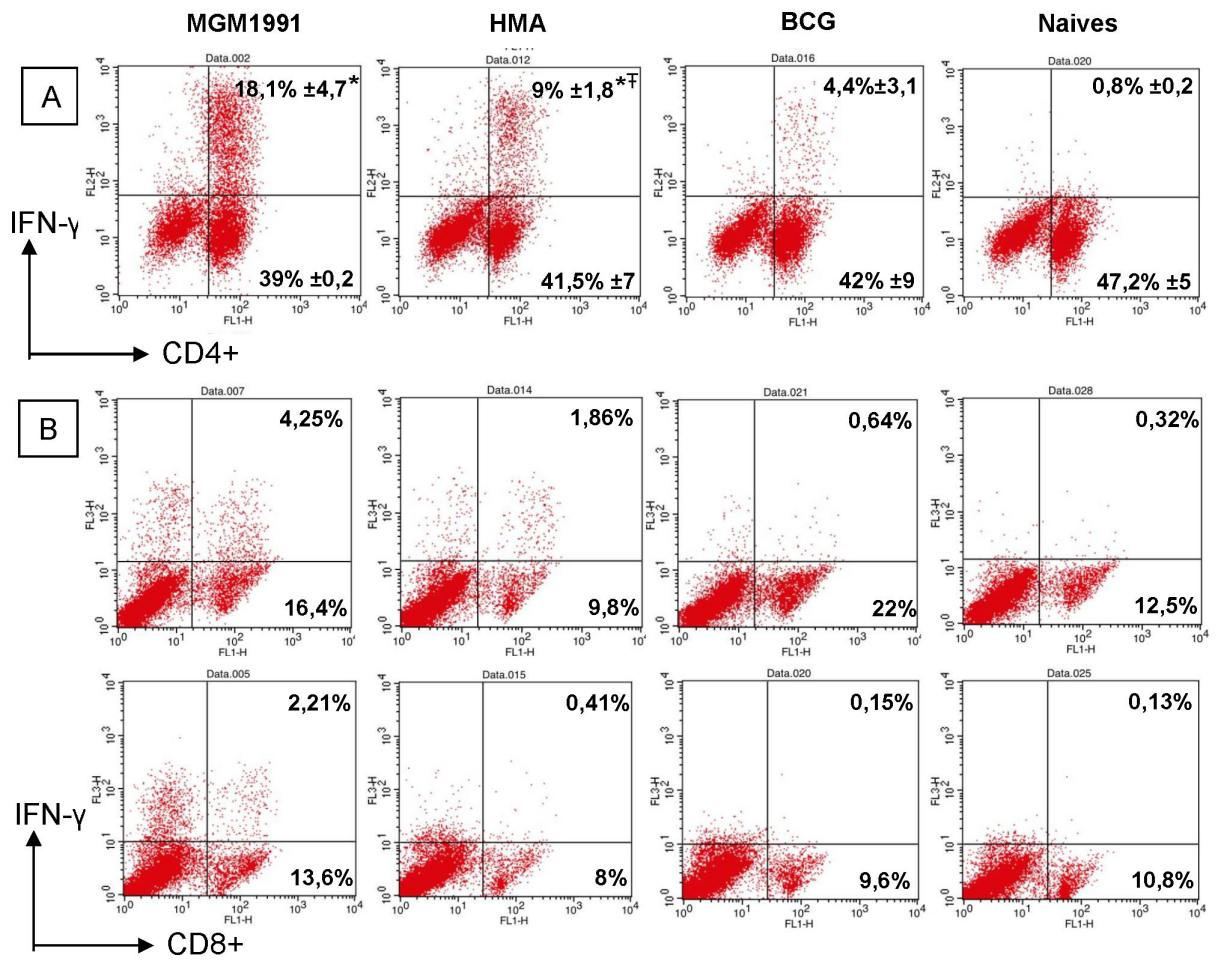
Mycobacteria specific intracellular IFN-γ production in CD4^+^ and CD8^+^ T spleen cells of mice vaccinated with MGM1991, HMA or BCG vaccine at 4 weeks after immunization. Upper panel (A): ICS staining of CD4^+^ spleen cells from C57BL/6 mice vaccinated 4 weeks before with 5x10^4^ CFU of MGM1991, HMA or *M. bovis* BCG by the subcutaneous route or from unvaccinated (naïve) mice. Mean % of positive cells in each quadrant (of 3 mice tested individually in each group). Lower 2 panels (B): ICS staining of CD8^+^ spleen cells from C57BL/6 mice vaccinated 4 weeks before with 5x10^4^ CFU of MGM1991, HMA or *M. bovis* BCG by the subcutaneous route or from unvaccinated (naïve) mice. Results show results obtained in 2 individual mice. ^*^ *p*<0,05 MGM1991 or HMA vs BCG; ^Ŧ^ *P*<0,05 MGM1991 vs HMA; (Mann-Whitney test).

**Figure 5:**
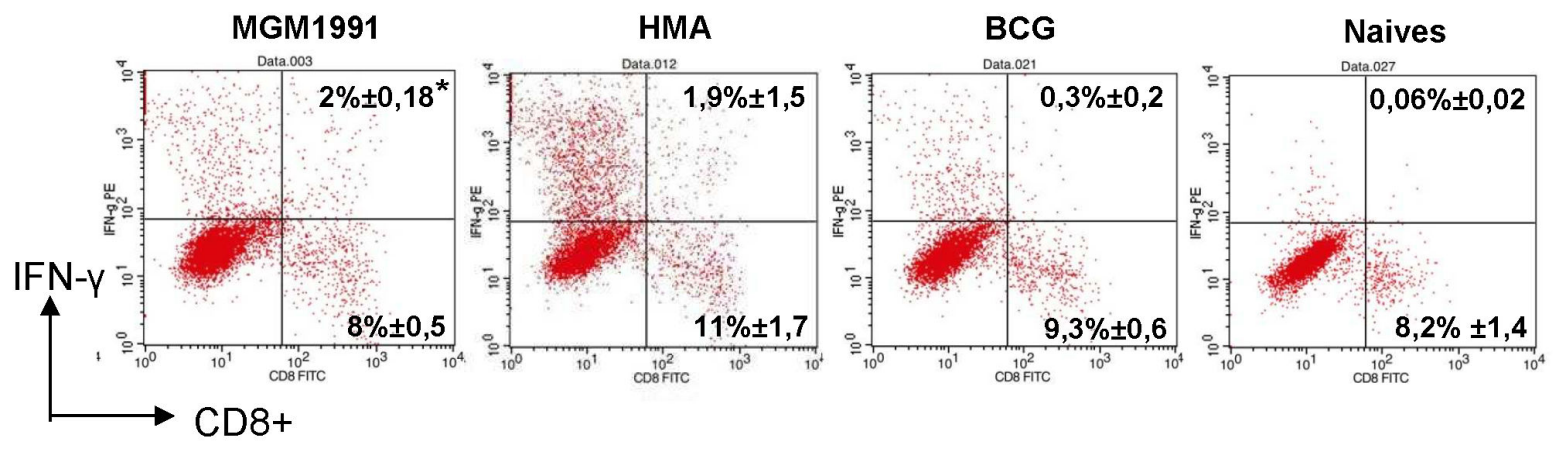
Mycobacteria specific intracellular IFN-γ production in CD8+ T spleen cells of mice vaccinated with MGM1991, HMA or BCG vaccine at 14 weeks after immunization. ICS staining of CD8+ spleen cells from C57BL/6 mice vaccinated 14 weeks before with 5x10^4^ CFU of MGM1991, HMA or *M. bovis* BCG by the subcutaneous route or from unvaccinated (naïve) mice. Mean % of positive cells in each quadrant (of 3 mice tested individually in each group). *p <0,05 MGM1991 vs BCG (Mann-Whitney test).

To investigate which T cell population was responsible for the increased IFN-γ response in mice vaccinated with the *M.tb* mutants, we performed an intracellular cytokine staining and flow cytometric analysis on CD4^+^ and CD8^+^ T cells from mice vaccinated with either *M.tb* mutant or BCG and from unvaccinated mice. About 4% of CD4^+^ T cells produced mycobacteria specific IFN-γ at 4 weeks post BCG vaccination following stimulation with *M.tb* culture filtrate (Figure **4**). This number increased to 9% in mice vaccinated with the HMA mutant and in animals vaccinated with MGM1991, the number of IFN-γ producing CD4^+^ T cells increased to 18% (p< 0.05). As expected, CD8^+^ IFN-γ producing cells were hardly detected upon BCG vaccination, but significant responses could be detected in animals vaccinated with *M.tb* mutant HMA and particularly MGM1991. Sustained increased levels of IFN-γ producing CD8^+^ T cells in animals vaccinated with the two mutants as compared to BCG, were still detected after 14 weeks of immunization (Figure **5**)

### Vaccination with *M.tuberculosis* MGM1991 or HMA mutants confers better protection against intratracheal challenge with virulent *M. tuberculosis* H37Rv than vaccination with BCG

**Figure 6:**
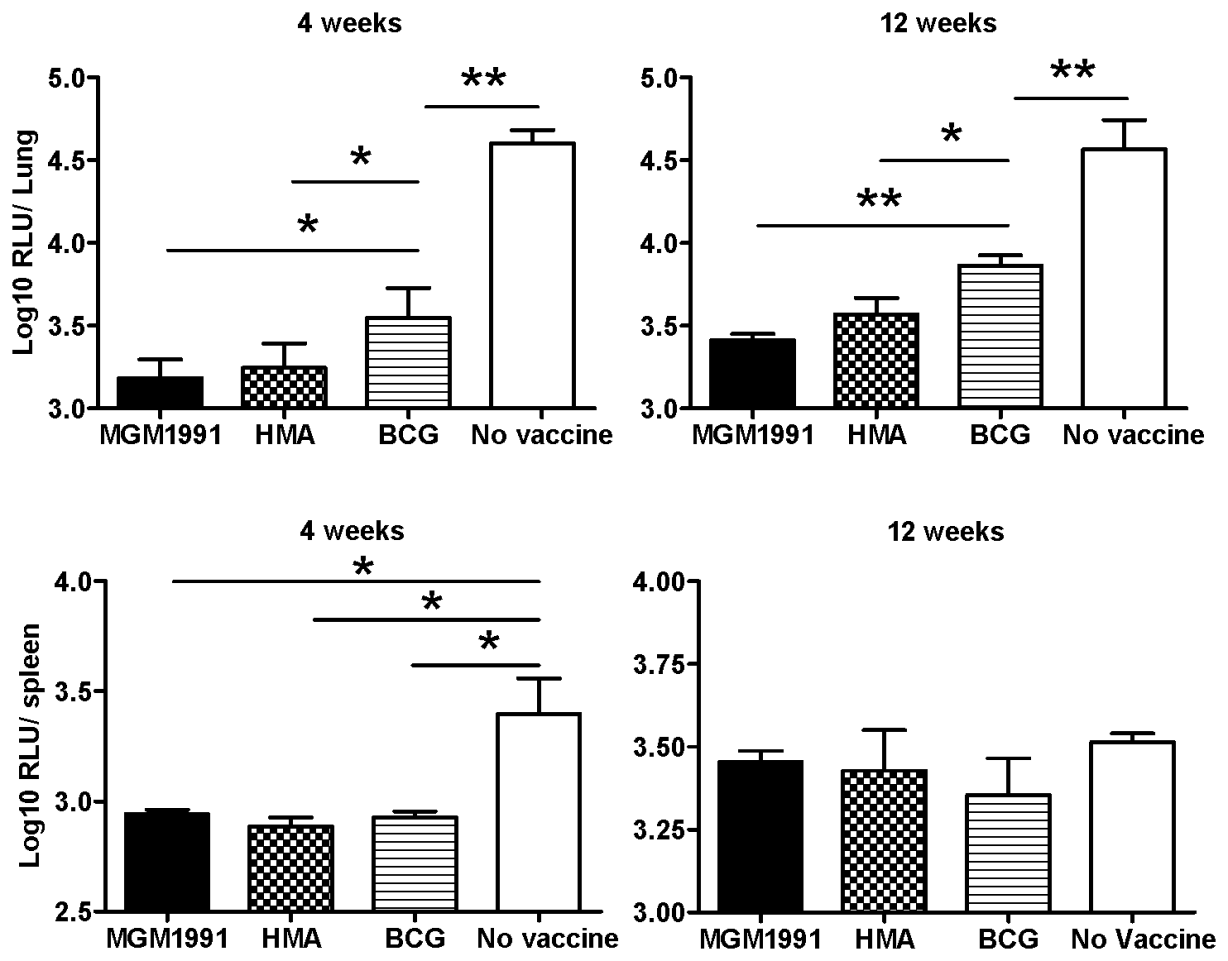
Bacterial replication in lungs and spleen of C57BL/6 mice vaccinate d with MGM1991, HMA or BCG and challenged with 10^4^ CFU of virulent luminescent *M. tuberculosis*. Mean number of bacteria in lungs and spleen of mice vaccinated with 5x10^4^ MGM1991, HMA or BCG or unvaccinated mice (naïve), challenged 12 weeks later with 10^4^ CFU of virulent *M.tb* by the intratracheal route and sacrificed 4 and 12 weeks after challenge. Results are reported as mean log_10_ relative light units (RLU) ± SD of 5 mice tested individually; * p < 0.05; ** p < 0.01 (Mann-Whitney test) .

Subcutaneous vaccination with 5x10^4^ CFU of the two *M.tb* mutants conferred strong protection against intratracheal challenge with a low dose 10^4^ CFU of virulent *M.tb* as indicated by reduced bacterial numbers in lungs both at 4 and 12 weeks post challenge (Figure **6**). At both time points, mutants conferred significantly more protection than the BCG vaccine. In spleen, both mutants and BCG conferred a significant and comparable protection at 4 weeks after challenge, whereas at 12 weeks no protection was detected in animals vaccinated with either of the three vaccines as bacterial numbers in spleen of naïve unvaccinated had also decreased, probably because of the low challenge dose used.

**Figure 7:**
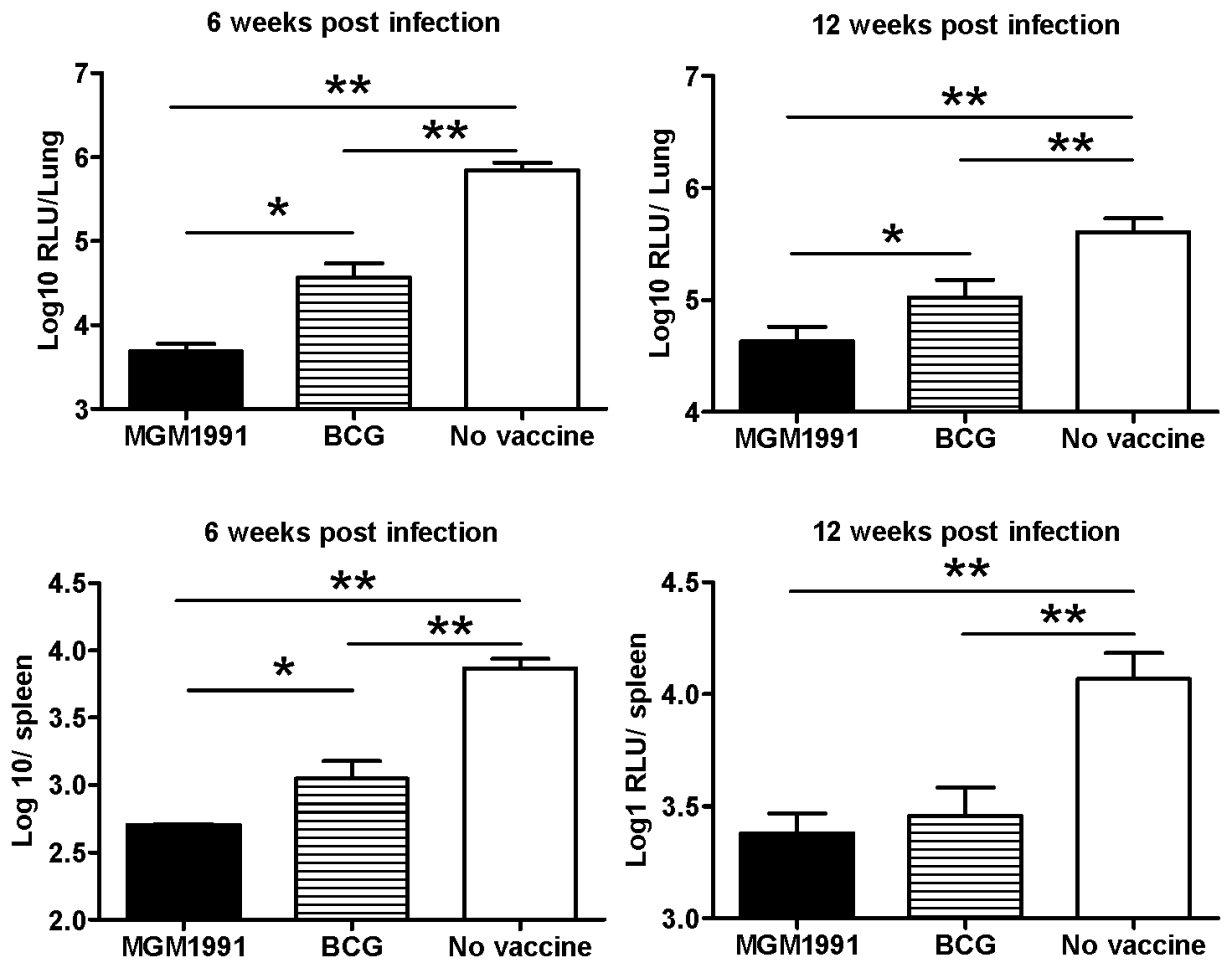
Bacterial replication in lungs and spleen of BALB/c mice vaccinated with MGM1991, or BCG and challenged with 10^5^ CFU of virulent luminescent *M. tuberculosis*. Mean number of bacteria in lungs and spleen of C57BL/6 mice vaccinated with 5x10^5^CFU of MGM1991 mutant or BCG or unvaccinated mice (naïve), challenged 12 weeks later with 10^5^CFU of virulent *M.tb* by the intratracheal route and sacrificed 6 or 12 weeks after challenge. Results are reported as mean log_10_ relative light units (RLU) ± SD of 5 mice tested individually; * *P* < 0.05; ** *P* < 0.01 (Mann-Whitney test).

**Figure 8:**
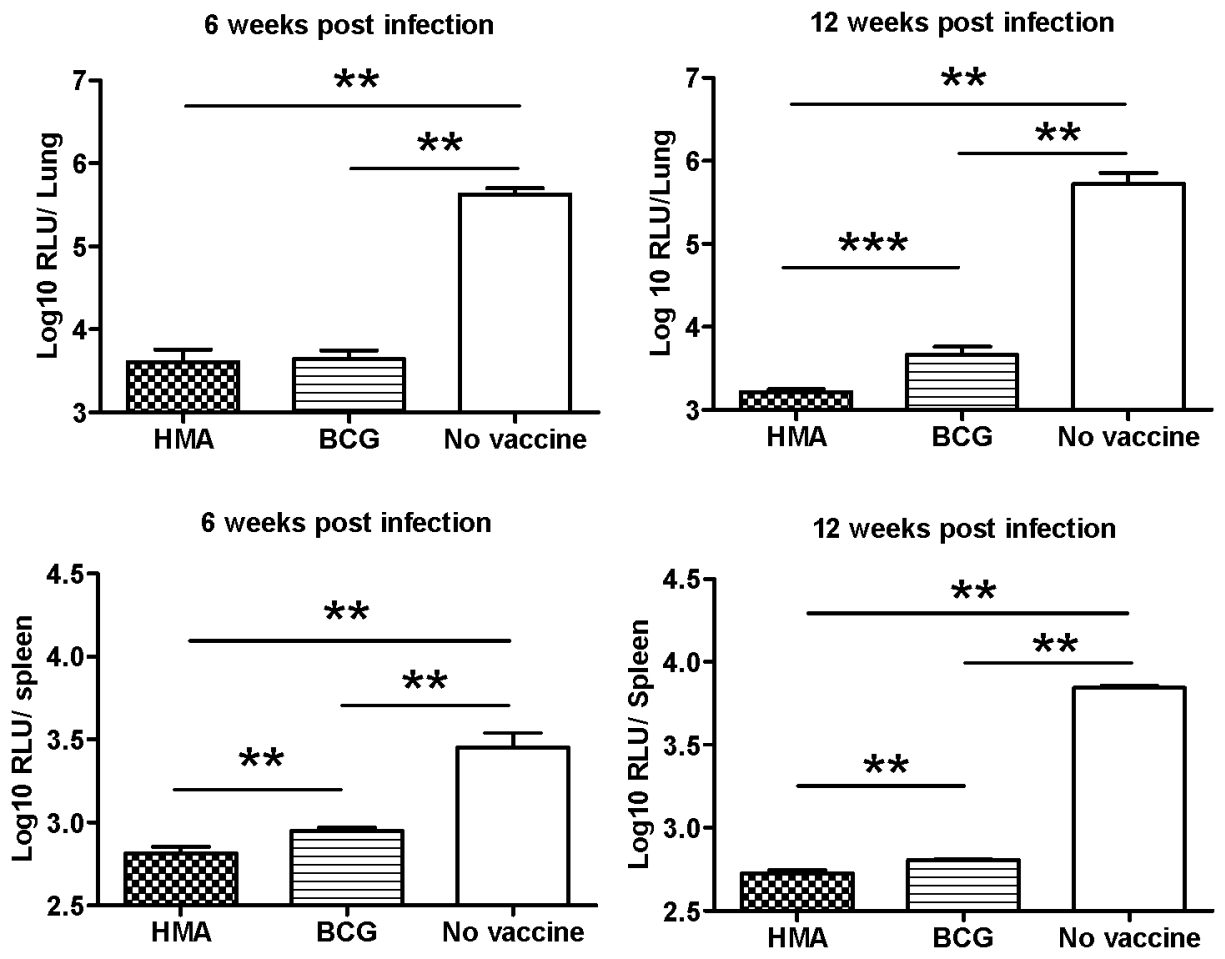
Bacterial replication in lungs and spleen of BALB/c mice vaccinated with HMA or BCG and challenged with 10^5^ CFU of virulent luminescent *M. tuberculosis*. Mean number of bacteria in lungs and spleen of BALB/c mice vaccinated with 5x10^5^CFU of HMA mutant or BCG or unvaccinated mice (naïve), challenged 12 weeks later with 10^5^CFU of virulent *M.tb* by the intratracheal route and sacrificed 6 or 12 weeks after challenge. Results are reported as mean log_10_ relative light units (RLU) ± SD of 5 mice tested individually; ** *P*<0,01; *** *P*<0.001 (Mann-Whitney test).

Vaccination of BALB/c mice with a tenfold higher dose of MGM1991, HMA or BCG (5x10^5^ CFU) and a tenfold higher challenge dose (10^5^ CFU) of *M.tb* H37Rv confirmed the stronger vaccine potential of the *M.tb* mutants as compared to BCG at the lung level (Figure **7** and Figure **8**) 

## Discussion

The *M. bovis* BCG vaccine can protect children against the extrapulmonary forms of tuberculosis, i.e. cerebral meningitis and miliary TB, but its protection against the classical pulmonary form of TB -which is often the result of a reactivation of a latent TB infection- is more variable [17]. The reasons for this weak efficacy of BCG are not exactly clear and many hypotheses have been advanced. Possibly there is a waning of efficacy over time of a vaccine generally administered to neonates. Three clinical trials, using recombinant fusion protein Ag85B-ESAT6 (Hyb1), recombinant fusion protein Mtb39-Mtb32 (M72) and Modified Vaccinia Ankara virus expressing Ag85A (MVA85A) as boosting regimen after BCG priming are actually testing this hypothesis [18]. Although MVA85A boosting leads to strongly increased Ag85A specific, IFN-γ producing CD4+ T cells responses in BCG vaccines [19], protection against tuberculosis could not be increased by this boosting regimen in infants in a rural area near Cape Town, South Africa [20]. Other reasons for the failure of the BCG vaccine may be its weak potential to induce immune responses against so-called latency antigens, which are more highly expressed *in vitro* by *M.tb* grown in hypoxic or nutrient starvation conditions (supposedly mimicking the physiological conditions in the lung granuloma) [21]. Finally, BCG vaccine was derived from an *M. bovis* isolate, and it has become clear that profound genetic differences exist between *M. bovis* and classical *M.tb* strains [22]. Moreover, the attenuation of *M. bovis* resulted in the deletion of large chromosome regions encoding amongst others (a.o.) the ESAT-6 protein, associated with virulence but at the same time an immunodominant and protective antigen of *M.tb*. Therefore, new live attenuated vaccines based on parental *M.tb* strains offer an interesting alternative. Promising in this respect is the MTBVAC vaccine, attenuated by the deletion of the virulence genes *phoP* and *fadD26*, for which a phase 1 clinical trial has started in October 2012. PhoP is a transcriptional regulator of *M.tb* and as a result of its deletion, MTBVAC has lost a.o. its capacity to secrete ESAT-6 [23] and the pro-apoptotic potential of the parental *M.tb* MT103 strain [24], potential dependent on the activation of caspase expression by the ESAT6 protein [25]. In contrast to sub-unit vaccines, live attenuated vaccines offer the advantage of inducing stronger memory and of having a broader antigenic repertoire. Recently, Sette and his group showed that this breadth may be essential for protection, as indicated by the fact that CD4^+^ T cell responses in individual healthy donors with a latent TB infection were directed against on average 24 different MHC class II restricted epitopes and that a total of 82 antigens were recognized by more than 10% of LTBI donors [26]. Obviously, safety issues have to be considered for live, attenuated vaccines and in 2005, WHO published the Geneva consensus on the use of live attenuated vaccines based on *M.tb* recommending at least two non-reverting independent mutations [27].

Here we have shown that two live attenuated *M.tb* mutants, affected in their mycolic acid composition and more specifically lacking all oxygenated mycolic acids, are promising new vaccine candidates as TB vaccines. Stronger Ag85A specific CD4^+^ Th1 cell responses were detected than upon vaccination with BCG. Moreover vaccination with the *M.tb* mutants but not with *M. bovis* BCG induced Th1 responses against the immunodominant ESAT-6 protein. Furthermore, besides CD4^+^ responses also mycobacteria specific CD8^+^ T cells were induced with the two *M.tb* mutants, in contrast to the BCG vaccine which showed, as expected, a very weak potential for generating MHC class I restricted responses. The induction of these CD8^+^ responses by the *M.tb* mutants, may be the result of an activation of caspase expression by ESAT-6 in infected macrophages and a subsequent generation of apoptotic vesicles leading to cross-priming events, as reported by Winau et al [28].A more detailed study of the pro-apoptotic potential and more in general of the innate immune responses induced by the *M.tb* mutants and comparison with the *M. bovis* BCG vaccine is needed to confirm this hypothesis (in progress).

 MGM1991 overall induced a stronger immune Th1 (IL-2 and IFN-γ and TNF-α) and IL-17A response than HMA mutant, but both mutants conferred comparable protection against *M.tb* challenge. It is possible that the parental background of the two mutants, i.e. Erdman vs. H37Rv is responsible for the stronger immunogenicity of MGM1991. On the other hand, HMA mutant still has the distal and proximal cyclopropanation of the α-mycolic acid and -as already mentioned- we and others have previously shown that cyclopropanation may have suppressive effects on inflammatory responses and the induction of IL-12p40 and TNF-α [8,29,30]. It must be emphasized that too strong inflammatory responses may have undesirable side effects and a correct balance between pro- and anti-inflammatory signals may be needed for optimal vaccine immunogenicity. The oxygenated MA-classes in particular seem to mediate pro-inflammatory cytokine release from macrophages and foam cell accumulation at the interface of host-pathogen interaction [31]. MGM1991 mutant produces only α-mycolate and *in vivo* this mutant triggers a weaker inflammatory response than wild type Erdman strain [8]. Using a series of synthetic mycolic acids, Vander Beken*et al* also showed that α-mycolic acid is inert *in vivo*, whereas oxygenated methoxy- and keto-MA with cis-cyclopropane stereochemistry elicited solid to mild inflammatory responses respectively [32]. In this respect, it is worth mentioning that the *M. bovis* BCG strain used in our study is a derivative of the Pasteur strain, which together with Glaxo, Prague , Danish and Chinese substrains is known to lack methoxy-mycolates [33].

It has been proposed that the attenuation of the *M.tb* HMA mutant is due to decreased permeability of the cell wall [9]. This loss of permeability could decrease the availability of specific nutrients, which could explain why these mutants grow more slowly and form smaller colonies when plated on solid medium such as 7H11 Middlebrook agar. Oxygenated mycolates are expected to confer a higher fluidity to the cell wall and mutants lacking these oxygenated species may have increased resistance to drugs. Also, mutant MGM1991 is more resistant to the detergent tyloxapol than mutant MGM1990 (lacking all cyclopropanation but producing both α- and oxygenated mycolic acids), indicating that loss of oxygenated lipids may indeed render *Mtb* more resistant to severe detergent stress [8]. In conclusion, we have shown that two *M.tb* mutants lacking oxygenated mycolic acids confer better protection against virulent *M.tb* than *M. bovis* BCG vaccine. In contrast to the MTBVAC vaccine which lacks the capacity to secrete ESAT-6 and is deficient in ESX-1 induced apoptosis [34], immune responses induced with the irreversibly attenuated mutants MGM1991 and HMA are directed against mycolyl transferase Ag85A but also against ESAT-6. Although not formally proven, the strong CD8^+^ T cell responses induced with MGM1991 and HMA are probably related to their capacity to secrete the pro-apoptotic virulence factor ESAT-6. Obviously, more work is needed to confirm their vaccine potential in *M.tb* infected guinea pigs and non-human primates.

## Supporting Information

Figure S1 **MGM1991 mutant shows attenuated phenotype as compared to wild type *M.tb* Erdman strain in intratracheally infected C57BL/6 and DBA/2 mice.** (A) C75BL/6 mice were infected by intratracheal (IT) route with 10^4^ CFU/mice of WT *M.tb* strain (Erdman) or MGM1991 *M.tb* strain and bacterial burden in lungs was evaluated at days 7, 21 and 70 after infection. * *P*<0.05 WT vs MGM1991 (Mann-Whitney test); (B) DBA2 mice survival after intratracheal infection with 10^5^ CFU/mice of WT *M.tb* strain (Erdman) or MGM1991 *M.tb* strain. Median survival time was 7 weeks for mice infected with WT *M.tb* strain versus 29 weeks for mice infected with MGM1991 *M.tb* strain. (** *P*< 0.005: Chi Square test).
(PPT)

Table S1 **Spleen cell IFN-γ production in C57BL/6 mice vaccinated with MGM1991, HMA or BCG vaccine 20 weeks before.** IFN-γ level in spleen cell culture supernatant of C57BL/6 mice vaccinated 20 weeks before with 5x10^4^ CFU of MGM1991, HMA or *M. bovis* BCG by the subcutaneous route or from unvaccinated (naïve) mice and stimulated in vitro with PPD or recombinant Ag85A (5 µg/ml) or with I-A^b^ restricted immunodominant peptides spanning aa 241-260 of Ag85A or aa 1-20 of ESAT-6 (10 µg/ml) or latency antigens Rv1733c, Rv2626c, Rv2627c, Rv2628. Cytokine levels are expressed in pg/ml (mean ± SD of 3 to 4 mice tested individually). ^Ψ^ *P*<0.05 MGM1991 vs HMA; ^*^ *P*<0.05 MGM1991 vs BCG; ^Ŧ^ *P*<0.05 MGM1991 vs Naïve (Mann-Whitney test).
(PPT)
