# Supplementary figures and images for: M.tuberculosis Mutants Lacking Oxygenated Mycolates Show Increased Immunogenicity and Protective Efficacy as Compared to M. bovis BCG Vaccine in an Experimental Mouse Model
Source: PLoS One. 2013 Oct 17;8(10):e76442. doi: 10.1371/journal.pone.0076442 (PMC3798287; doi:10.1371/journal.pone.0076442)

## Slide 1
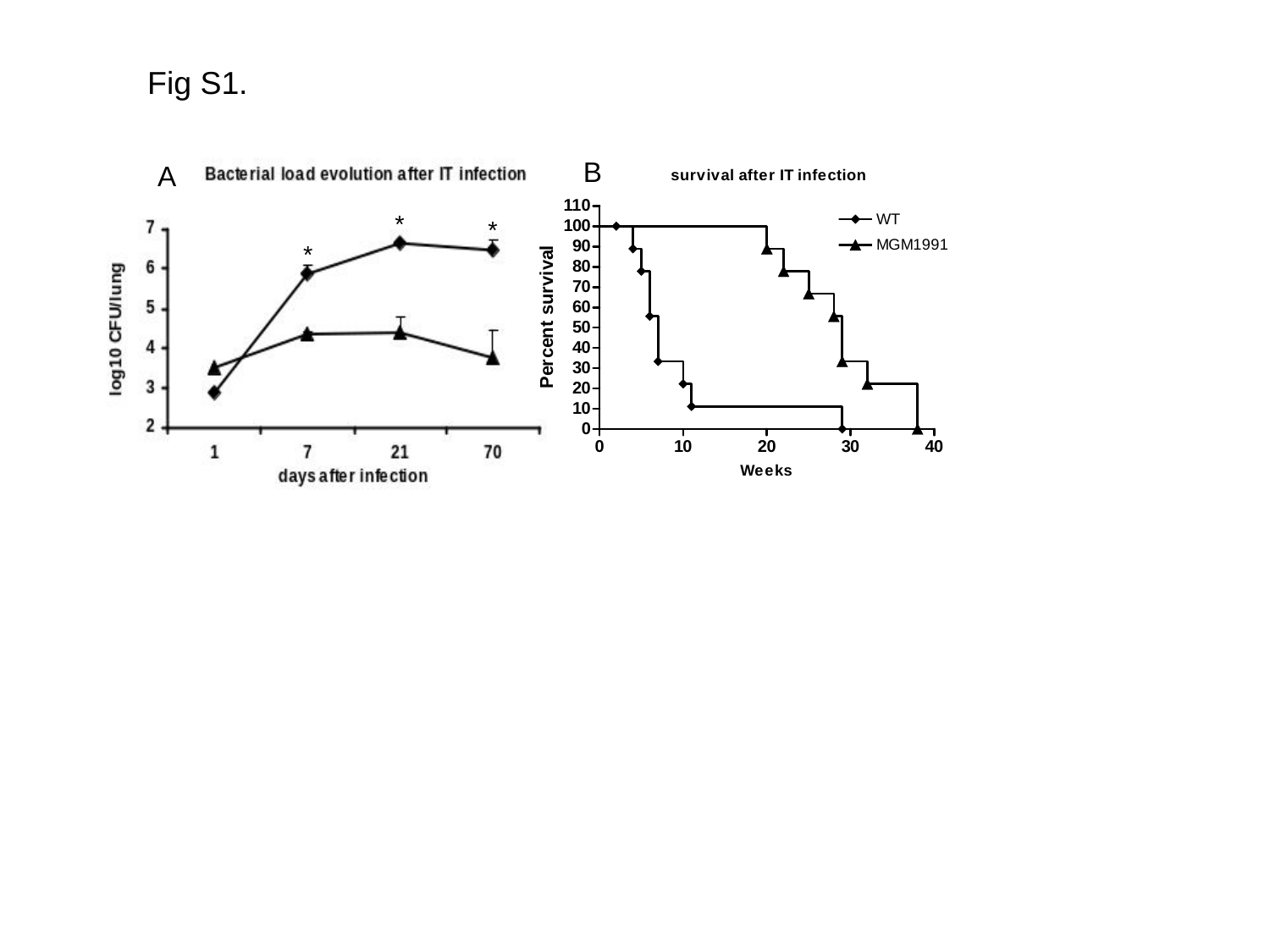

Fig S1.
*
*
*
B
A

Supplement: Figure S1 — MGM1991 mutant shows attenuated phenotype as compared to wild type M.tb Erdman strain in intratracheally infected C57BL/6 and DBA/2 mice. (A) C75BL/6 mice were infected by intratracheal (IT) route with 104 CFU/mice of WT M.tb strain (Erdman) or MGM1991 M.tb strain and bacterial burden in lungs was evaluated at days 7, 21 and 70 after infection. * P<0.05 WT vs MGM1991 (Mann-Whitney test); (B) DBA2 mice survival after intratracheal infection with 105 CFU/mice of WT M.tb strain (Erdman) or MGM1991 M.tb strain. Median survival time was 7 weeks for mice infected with WT M.tb strain versus 29 weeks for mice infected with MGM1991 M.tb strain. (** P< 0.005: Chi Square test). (PPT) [file pone.0076442.s001.ppt]
