# Supplementary material for: M.tuberculosis Mutants Lacking Oxygenated Mycolates Show Increased Immunogenicity and Protective Efficacy as Compared to M. bovis BCG Vaccine in an Experimental Mouse Model
Source: PLoS One. 2013 Oct 17;8(10):e76442. doi: 10.1371/journal.pone.0076442 (PMC3798287; doi:10.1371/journal.pone.0076442)
Supplement: Table S1 — Spleen cell IFN-γ production in C57BL/6 mice vaccinated with MGM1991, HMA or BCG vaccine 20 weeks before. IFN-γ level in spleen cell culture supernatant of C57BL/6 mice vaccinated 20 weeks before with 5x10^4^ CFU of MGM1991, HMA or M. bovis BCG by the subcutaneous route or from unvaccinated (naïve) mice and stimulated in vitro with PPD or recombinant Ag85A (5 µg/ml) or with I-Ab restricted immunodominant peptides spanning aa 241-260 of Ag85A or aa 1-20 of ESAT-6 (10 µg/ml) or latency antigens Rv1733c, Rv2626c, Rv2627c, Rv2628. Cytokine levels are expressed in pg/ml (mean ± SD of 3 to 4 mice tested individually). Ψ P<0.05 MGM1991 vs HMA; * P<0.05 MGM1991 vs BCG; Ŧ P<0.05 MGM1991 vs Naïve (Mann-Whitney test). (PPT) [file pone.0076442.s002.ppt]

## Slide 1
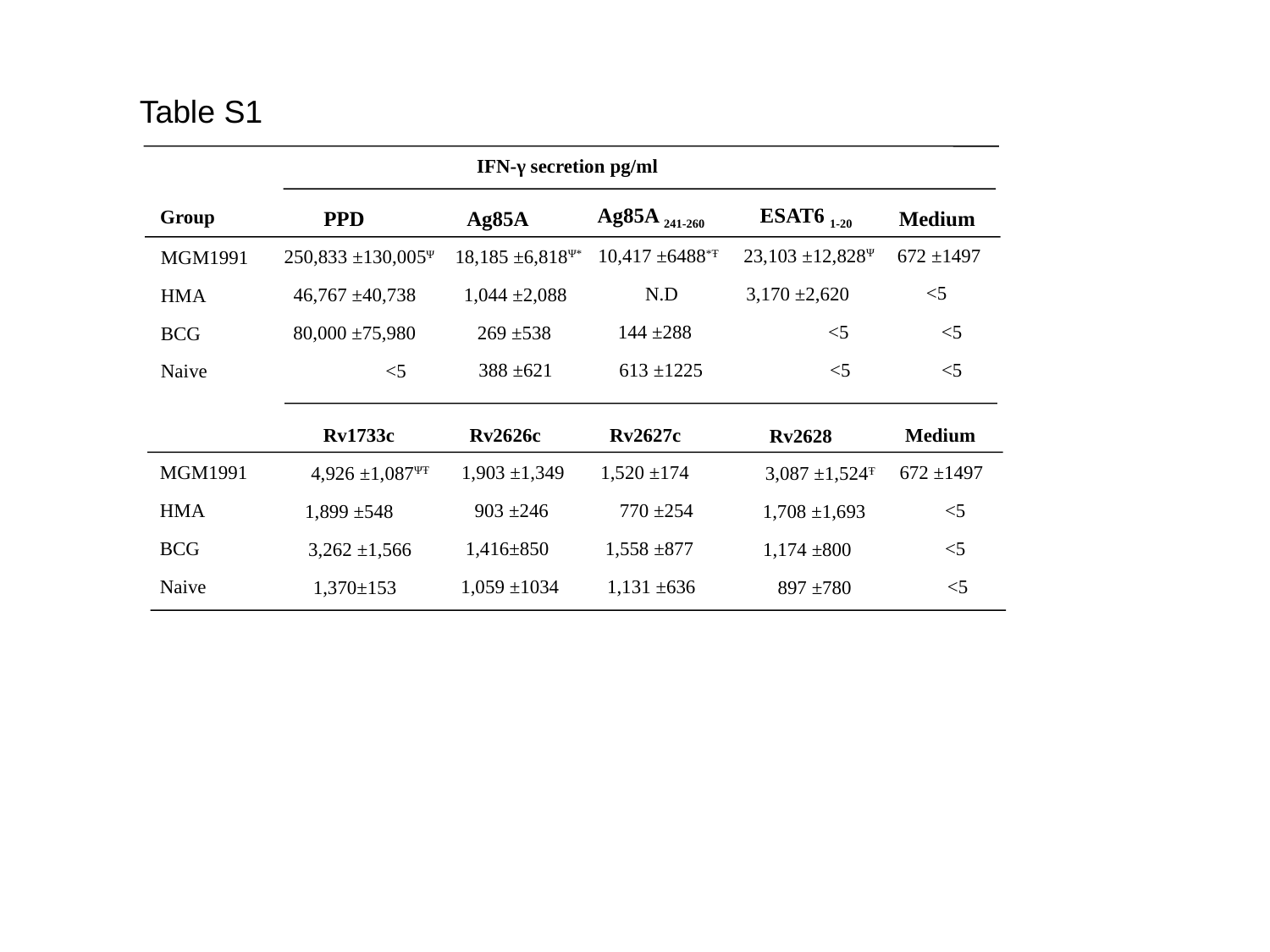

Table S1
IFN-γ secretion pg/ml
Ag85A
Ag85A 241-260
ESAT6 1-20
Medium
PPD
Group
 23,103 ±12,828Ψ
672 ±1497
18,185 ±6,818Ψ*
10,417 ±6488*Ŧ
250,833 ±130,005Ψ
MGM1991
<5
N.D
3,170 ±2,620
1,044 ±2,088
46,767 ±40,738
HMA
144 ±288
<5
<5
269 ±538
80,000 ±75,980
BCG
613 ±1225
<5
<5
388 ±621
<5
Naive
Rv2626c
Rv2627c
Medium
Rv1733c
Rv2628
MGM1991
1,903 ±1,349
1,520 ±174
672 ±1497
4,926 ±1,087ΨŦ
3,087 ±1,524Ŧ
HMA
903 ±246
770 ±254
<5
1,899 ±548
1,708 ±1,693
BCG
1,416±850
1,558 ±877
<5
3,262 ±1,566
1,174 ±800
Naive
 1,059 ±1034
1,131 ±636
<5
1,370±153
897 ±780
